# Neuroprotective Effects of α-Tocotrienol on Kainic Acid-Induced Neurotoxicity in Organotypic Hippocampal Slice Cultures

**DOI:** 10.3390/ijms140918256

**Published:** 2013-09-05

**Authors:** Na Young Jung, Kyung Hee Lee, Ran Won, Bae Hwan Lee

**Affiliations:** 1 Department of Physiology, Brain Korea 21 Project for Medical Science, Yonsei University College of Medicine, Seoul 120-752, Korea; E-Mail: sci04@naver.com; 2 Department of Dental Hygiene, Division of Health Science, Dongseo University, Busan 617-716, Korea; E-Mail: kyhee@gdsu.dongseo.ac.kr; 3 Department of Biomedical Laboratory Science, Division of Health Science, Dongseo University, Busan 617-716, Korea; E-Mail: wonran@gdsu.dongseo.ac.kr

**Keywords:** kainic acid, organotypic hippocampal slice culture, antioxidant, alpha-tocopherol, alpha-tocotrienol

## Abstract

Vitamin E, such as alpha-tocopherol (ATPH) and alpha-tocotrienol (ATTN), is a chain-breaking antioxidant that prevents the chain propagation step during lipid peroxidation. In the present study, we investigated the effects of ATTN on KA-induced neuronal death using organotypic hippocampal slice culture (OHSC) and compared the neuroprotective effects of ATTN and ATPH. After 15 h KA (5 μM) treatment, delayed neuronal death was detected in the CA3 region and reactive oxygen species (ROS) formation and lipid peroxidation were also increased. Both co-treatment and post-treatment of ATPH (100 μM) or ATTN (100 μM) significantly increased the cell survival and reduced the number of TUNEL-positive cells in the CA3 region. Increased dichlorofluorescein (DCF) fluorescence and levels of thiobarbiturate reactive substances (TBARS) were decreased by ATPH and ATTN treatment. These data suggest that ATPH and ATTN treatment have protective effects on KA-induced cell death in OHSC. ATTN treatment tended to be more effective than ATPH treatment, even though there was no significant difference between ATPH and ATTN in co-treatment or post-treatment.

## 1. Introduction

Kainic acid (KA), an agonist for kainate and alpha-amino-3-hydroxy-5-methyl-4-isoxazolepropionic acid (AMPA) receptors, is an excitatory neurotoxic substance. KA can induce characteristic limbic seizures and selective neuronal cell death in both hippocampal CA1 and CA3 regions. The mechanisms involved in this pathogenesis appear to be linked to oxidative stress. KA activates the glutamate receptor and induces a transmembrane ion imbalance by calcium influx, and then generates the reactive oxygen species (ROS). ROS attacks macromolecules within neurons, resulting in membrane lipid peroxidation, and structural and functional changes in proteins and DNA strand breaks [1,2]. The brain is highly susceptible to oxidative stress because of its high consumption of oxygen energy and glucose, large amount of peroxidizable polyunsaturated fatty acid, and relatively low antioxidant capability [3]. Increased production of free radicals may play a key role in the neuronal injury caused by KA-induced lipid peroxidation. Therefore, scavenging or reducing the KA-induced free radicals is important for preventing the harmful insults resulting from KA treatment [4,5].

Vitamin E is the term used for a group of tocopherols and tocotrienols, each with alpha, beta, gamma and delta analogs, that are essential components of biological membranes where they have both antioxidant and non-antioxidant functions [6]. Vitamin E is a chain-breaking antioxidant; it is able to repair oxidizing radicals directly, preventing the chain propagation step during lipid peroxidation [7]. In the vitamin E group, alpha-tocopherol (ATPH) and alpha-tocotrienol (ATTN) are suggested to have the highest biological activity [8,9]. However, the functions of tocotrienols have not been fully elucidated. Consistent with the antioxidative effects of tocotrienols, tocotrienol supplementation reduces blood levels of lipid peroxides with an improved blood flow in patients with carotid atherosclerosis [10]. On the other hand, several reports have suggested that tocotrienols possess non-antioxidant functions that contribute to their cholesterol-lowering, anticarcinogenic and cytoprotective properties [11–13].

However, little is known about the effects of tocotrienols on CNS neurons even though there were several early reports, which showed a protective role for alpha-tocopherol or vitamin E [14,15]. Organotypic hippocampal slice culture (OHSC) models represent a useful intermediate tool for studying chronic, progressive cell damage [16]. OHSC models maintain internal structure and function as observed *in vivo*, but allow an examination of the direct effects of excitotoxic compounds on the hippocampus that are not possible to study *in vivo*. In this study, we investigated whether KA-induced neuronal death in OHSC can be prevented by ATTN by measuring PI uptake, ROS generation, and lipid peroxidation levels and compared the neuroprotective effects of ATTN and ATPH.

## 2. Results and Discussion

### 2.1. Effects of ATPH and ATTN on KA-Induced Neuronal Toxicity in OHSC

**Figure 1:**
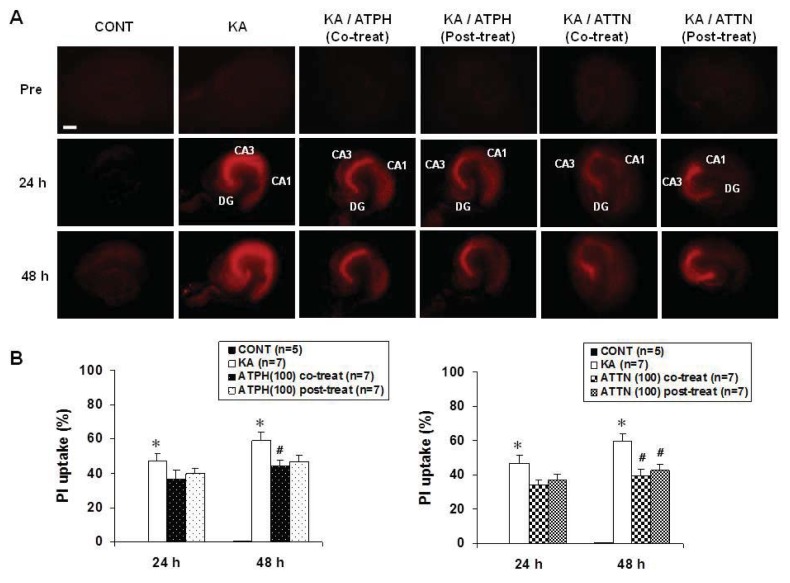
Effects of alpha-tocopherol (ATPH) and alpha-tocotrienol (ATTN) on KA-induced PI uptake in organotypic hippocampal slice culture (OHSC). Representative PI uptake images from CONT (untreated), KA (5 μM KA only treated), KA/ATPH Co-treat (100 μM ATPH with KA), KA/ATPH Post-treat (100 μM ATPH after KA), KA/ATTN Co-treat (100 μM ATTN with KA), and KA/ATTN Post-treat (100 μM ATTN after KA) slices (**A**). Quantification of PI images. Data are presented as means ± S.E.M. of 5 to 7 experiments (**B**). Asterisks (*) indicate statistically significant difference from control (* *p* < 0.001); Sharps (^#^) indicate statistically significant difference from KA-treated cultures (^#^*p* < 0.05). Scale bar: 200 μm.

PI uptake was measured to assess the effects of ATPH and ATTN on KA-induced cell death. When hippocampal slices were exposed to 5 μM KA for 15 h, the PI uptake in the CA3 region was significantly higher than that in the CA1 region (Figure 1A). Treatment with ATPH significantly reduced the PI uptake at 48 h after treatment as compared to post-treatment with ATPH. Treatment with ATTN also showed significantly reduced PI uptake in both co- and post- treatment at 48 h after treatment. Thus, co-treatment using ATPH (100 μM) or ATTN (100 μM) with KA significantly reduced the level of PI uptake in the CA3 region compared with KA treatment alone (Figure 1B). Further, the same trend was also observed with post-treatment using ATPH or ATTN 15 h after KA treatment.

### 2.2. Cresyl Violet Staining and TUNEL Assay

**Figure 2:**
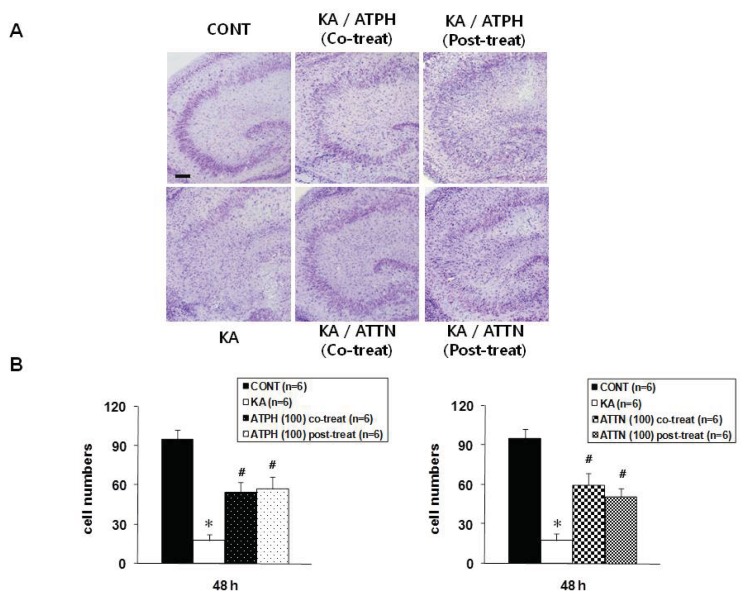

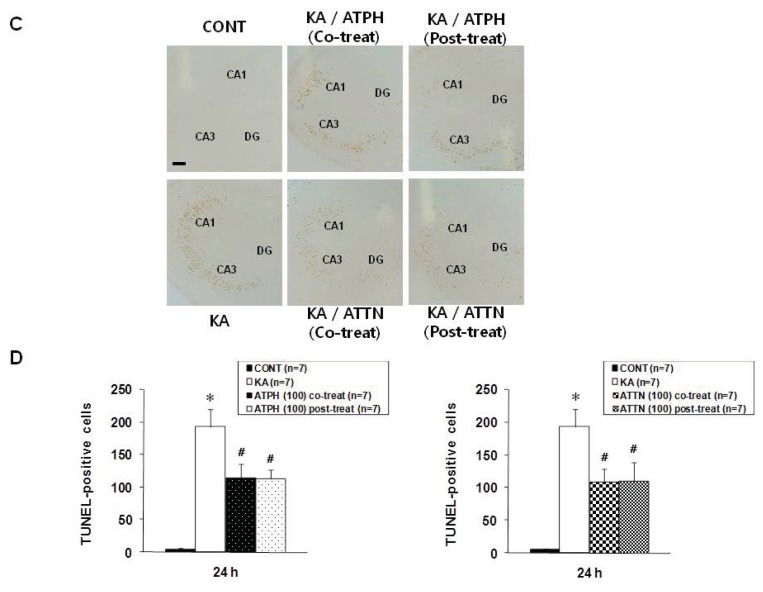
Cresyl violet and TUNEL staining in the CA3 region of OHSC. Cresyl violet staining was performed at the end of 48 h recovery after KA treatment for 15 h. Representative images from CONT (untreated), KA (5 μM KA only treated), KA/ATPH Co-treat (100 μM ATPH with KA), KA/ATPH Post-treat (100 μM ATPH after KA), KA/ATTN Co-treat (100 μM ATTN with KA), and KA/ATTN Post-treat (100 μM ATTN after KA) slices (**A** and **C**). Cresyl violet-positive cells regarded as survival cells and TUNEL-positive cells indicated as apoptotic cell death were quantified in CA3 region (**B** and **D**). Data are presented as means ± S.E.M. of 6 experiments for cresyl violet and 5 experiments for TUNEL staining. Asterisks (*) indicate statistically significant difference from control (* *p* < 0.001 in (**B**) and * *p* < 0.001 in (**D**)); Sharps (^#^) indicate statistically significant difference from KA-treated cultures (^#^*p* < 0.05). Scale bar: 200 μm.

Slices were stained with cresyl violet in order to measure cell survival and to determine the agreement with results from PI and a terminal deoxynucleotidyl transferase-mediated dUTP nickend labeling (TUNEL) staining. As shown in Figure 2A, the cresyl violet stained sections showed the KA-induced neuronal death was concentrated in the CA3 pyramidal neurons. However, cell death was significantly prevented by treatment with ATTP or ATTN (100 μM) compared to treatment with KA alone, although there were no statistically significant differences between ATTP and ATTN treatment for either co-treatment or post-treatment (Figure 2B).

To detect DNA fragmentation, TUNEL staining was performed 24 h after KA (5 μM) treatment for 15 h. KA significantly increased the number of TUNEL-positive cells in the CA3 region compared to the control, untreated slice, while 100 μM ATPH and ATTN blocked the response (Figure 2C). With both co- and post-treatment with ATPH and KA, the KA-induced neuronal death was significantly reduced, even though co-treatment and post-treatment results did not significantly differ. Similar to results obtained with ATPH treatment, ATTN treatment also significantly reduced the neuronal death with both co- and post-treatment as compared with KA only treatment (Figure 2D). The pattern of PI uptake corresponded with the distribution of cell survival detected by cresyl violet staining (Figure 2A) and TUNEL staining (Figure 2C).

### 2.3. Formation of ROS in KA-Induced Toxicity

**Figure 3:**
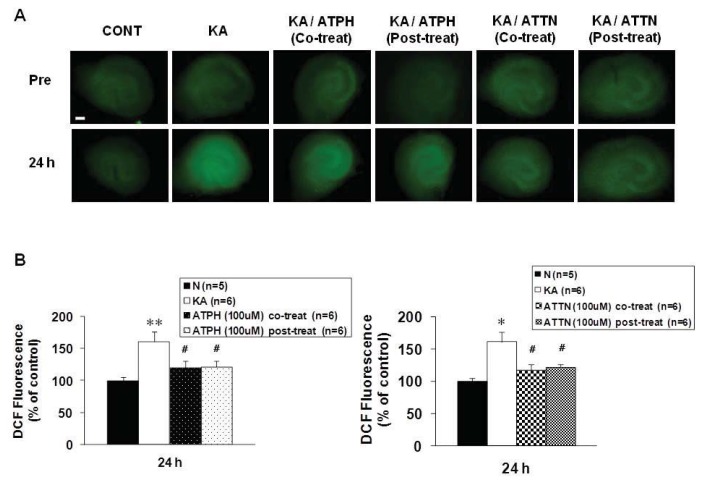
Effects of ATPH and ATTN on KA-induced increase in DCF fluorescence in OHSC. (**A**) Representative images from CONT (untreated), KA (5 μM KA only treated), KA/ATPH Co-treat (100 μM ATPH with KA), KA/ATPH Post-treat (100 μM ATPH after KA), KA/ATTN Co-treat (100 μM ATTN with KA), and KA/ATTN Post-treat (100 μM ATTN after KA) slices; (**B**) Quantification of DCF intensity. Data are expressed as percentage of control values and are presented as means ± S.E.M. of 5 to 6 experiments. Asterisks (*) indicate statistically significant difference from control (* *p* < 0.05, ** *p* < 0.001); Sharps (^#^) indicate statistically significant difference from KA-treated cultures (^#^*p* < 0.05). Scale bar: 200 μm.

We measured ROS accumulation at the end of the 24-h recovery after KA (5 μM) treatment using the fluorescence probe 2′,7′-dichlorofluorescein (DCF), which detects intracellular peroxides. Exposure of cultures to KA caused a significant increase in DCF fluorescence in all regions of the OHSC compared to control (Figure 3A). 100 μM ATPH or ATTN significantly decreased the fluorescence, while there were no statistical differences between co-and post-treatment (Figure 3B). The patterns of fluorescent images obtained from PI uptake and DCF staining were different. After 24 h of recovery following KA treatment, PI fluorescence was clearly evident in the CA3 region, whereas the patterns of DCF were more diffuse throughout the whole slice.

### 2.4. Attenuation of KA-Induced Lipid Peroxidation

**Figure 4:**
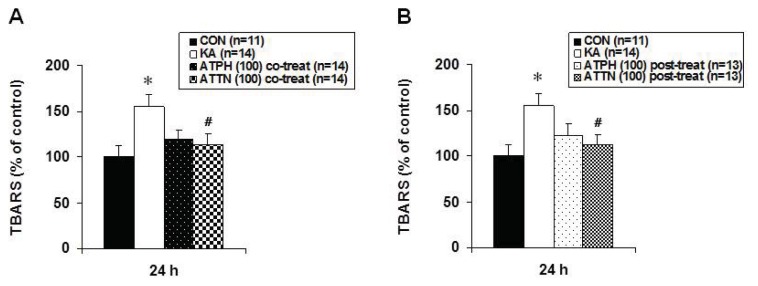
Effects of ATPH (**A**) and ATTN (**B**) on KA-induced increase in lipid peroxidation in OHSC. Lipid peroxidation was measured at 24 h recovery time after drugs treatment by TBARS assay as described in Methods. Results are expressed as percentage of control values and are means ± S.E.M. of 9 to 12 experiments. Asterisks (*) indicate statistically significant difference from control (* *p* < 0.05); Sharps (^#^) indicate statistically significant difference from KA-treated cultures (^#^*p* < 0.05).

The extent of lipid peroxidation was determined by the concentration of malondialdehyde (MDA), which is one of the end products of lipid peroxidation measured by the Thiobarbituric acid reactive substances (TBARS) assay. In KA-treated cultures, the levels of MDA were significantly elevated relative to those in the controls (Figure 4). After ATPH or ATTN co-treatment or post-treatment with KA, MDA levels tended to be lower compared to those in cultures treated with KA only. Moreover, in co-treatment or post-treatment of ATPH *versus* ATTN, although both showed a tendency to reduce MDA levels, ATTN showed statistically significant reduction in the level of MDA.

### 2.5. Discussion

KA treatment with OHSC has been used as an *in vitro* model to study the mechanisms of status epilepticus (SE)-induced neuronal damage and epileptogenesis, since KA treatment of slices induces region-specific neuronal death and reorganization of hippocampal circuitry [17,18]. In OHSC, many of the excitatory and inhibitory synapses of the transverse hippocampal slice are preserved [19,20]. KA induces cell loss in OHSC in a dose-dependent manner [21]. Low concentrations of KA result in a specific loss of CA3 neurons, while high concentrations induce complete neuronal death. In our previous study [22], we observed the same phenomenon of selective vulnerability in the CA3 region at 5 μM (low concentration) KA exposure using PI uptake, and the results of cresyl violet and TUNEL staining corresponded with the PI results. Neuronal cell death may be caused by excessive ROS, mitochondrial dysfunction and caspase activation. Cell death commonly occurs by necrosis or apoptosis, although this concept has recently been challenged [23]. Apoptosis is well known to be related to increased ROS generation and neurodegeneration.

Previous studies have shown a time-dependent increase in DCF fluorescence during treatment with excitotoxic compounds, with a peak at 0 h recovery after drug exposure that reduces with time [5,24]. We observed an increase in DCF fluorescence, which was consistent with the notion that ROS accumulation is a cause rather than a consequence of neuronal death. In agreement with these results, KA treatment caused a significant increase in DCF fluorescence levels, even at different time points. We assessed the production of ROS at 24 h after KA treatment for 15 h. We found that 100 μM of ATPH or ATTN significantly inhibited ROS formation compared to KA only-treated cultures.

Lipid peroxidation by ROS is known to be involved in the damaging mechanism of several acute and chronic brain disorders. The most prominent and currently used assay for lipid peroxidation is the TBARS assay. It is based on the reactivity of an end product of lipid peroxidation; MDA reacts with TBA to produce a red adduct. In ischemic and epileptic studies, lipid peroxidation levels have been shown to increase in parts of the brain such as the hippocampus, striatum, and cerebellum [25,26]. In this study, lipid peroxidation levels increased after KA treatment, and this was reduced by ATPH or ATTN treatment. Previous studies have shown the disagreement between DCF and TBARS data at peak times. DCF fluorescence peaked earlier than lipid peroxidation. This may indicate that lipid peroxidation is downstream from ROS generation [27]. Though this mechanism was not exactly confirmed by this study, the ability of ATPH and ATTN to inhibit the KA-induced increases in these parameters indicates that both ROS formation and MDA release occur downstream of free radical production induced by KA.

ATPH has been found to be effective against ferrous chloride seizures, hyperbaric oxygen seizures, and penicillin-induced seizures, where ROS production may have a role in the development of seizures itself [28,29]. Vitamin E primarily functions as an antioxidant that reacts with fatty acid peroxyl radicals produced from lipid peroxidation. Direct comparisons between the various tocopherol and tocotrienol isoforms have shown large differences in antioxidant activity. For many years, ATPH was considered the most potent antioxidant against lipid peroxidation in the vitamin E group [30]. Recently, however, there has been considerable discrepancy in its relative antioxidant effectiveness when compared to other isomers. On one hand, gamma-tocopherol was found to be more potent than ATPH particularly in its interaction with reactive nitrogen oxide species [31]. On the other hand, ATTN was also found to be a better antioxidant than ATPH [32,33]. Notably, Serbinova *et al.* [32] observed a remarkably higher anti-oxidant activity with tocotrienol against lipid peroxidation in rat liver microsomes than with ATPH. Kamat and Devasagayam [34] reported similar results in rat brain mitochondria and noted a stronger effect with gamma-tocotrienol. The α-isoforms of tocopherol and tocotrienol are the most potent lipid soluble antioxidants due to the level of methylation of the chromanol ring, which greatly improves the reactivity of the hydroxyl group and facilitates the transfer of the hydrogen to a peroxyl radical to form a tocopheroxyl or tocotrienoxyl radical [35]. Tocopherols and tocotrienols can be restored by reduction of tocopheroxyl and tocotrienoxyl radical by other antioxidants such as vitamin C and ubiquinol [36,37]. The antioxidant potencies of tocopherols and tocotrienols have been compared in a variety of extensive *in vitro* studies, and the results showed that tocotrienols have a higher free radical scavenging efficiency than tocopherols. Moreover, there are a limited number of studies about tocotrienols as an antioxidant *in vivo*, and also many studies have shown the effects of pre-treatment with vitamin E analogs. In the present study, we investigated the effects of ATPH and ATTN on KA-induced neuronal death in OHSC. There was a marked difference in the time course of these treatments as compared with previous studies. 100 μM ATPH and ATTN treatment dramatically protected pyramidal cells of CA3 against KA treatment, as based on results from PI uptake, cresyl violet staining, and TUNEL assay. In addition, ATPH and ATTN attenuated KA-induced ROS generation and lipid peroxidation. ATTN tends to have a more protective effect than ATPH on KA-induced neurotoxicity even though there was significant difference between ATPH and ATTN in co-treatment or post-treatment.

## 3. Experimental Section

### 3.1. Organotypic Slice Culture

All animal experiments were approved by the Institutional Animal Care and Use Committee of Yonsei University Health System. OHSC was conducted in accordance with our previous experimental protocols [5,18,22,24] modified from Stoppini *et al*. [38]. In brief, we rapidly removed hippocampi from seven-day-old Sprague-Dawley rat pups and transferred them to cold dissection media comprised of Gey’s balanced salt solution (Sigma, St. Louis, MO, USA) with 0.5% glucose and 3 mM KCl. Sections were cut to 350 μm with a McIlwain tissue chopper (Vibratome, O’Fallon, MO, USA) and inspected and cut into slices under a dissection microscope. After inspection, six slices were transferred onto a Millicell-CM membrane insert (Millipore, Billreka, MA, USA) set on a 6-well plate in 1 mL of culture medium composed of 50% Opti-MEM, 25% Hank’s balanced salt solution (HBSS), 25% heat-inactivated horse serum, 6.5 mg/mL d-glucose (AMRESCO Inc., Solon, OH, USA), pH 7.2 (all from Gibco BRL, Grand Island, NY, USA). Plates were kept in a 35 °C humidified incubator with 5% CO_2_. The media was changed three times a week. Slices were grown for 20–24 days *in vitro* (DIV) prior to drug treatment.

### 3.2. Drug Treatment and Assessment of Neuronal Injury

KA (5 μM) was applied for 15 h in OHSC. Stock solutions (10^3^× working concentration, 100 mM) of α-tocopherol (ATPH, Sigma, St. Louis, MO, USA) and α-tocotrienol (ATTN, Tocomin^®^, Kind gift from Carotech Berhad, Perak, Malaysia) were prepared in ethanol. Co-treatment was performed ATPH (100 μM) or ATTN (100 μM) were simultaneously added to the culture dishes with KA for 15 h (co-treatment) and changed culture medium obtained ATPH or ATTN. The post-treatment was conducted the ATPH or ATTN was treated for 24 h and 48 h after withdraw of the KA treatment for 15 h. These concentrations were chosen based on the maximal effects obtained in preliminary tests. For detection of neuronal cell death, cellular uptake of the fluorescent dye propidium iodide (PI, Sigma, St. Louis, MO, USA) was recorded. PI is basically nontoxic to neurons and has been used as an indicator of neuronal membrane and cell damage. PI was added to the culture medium to achieve a final concentration of 5 μg/mL. PI uptake was recorded by fluorescence microscopy (IX-71, Olympus, Tokyo, Japan) using a standard rhodamine filter and digital camera and then analyzed with the MetaMorph Imaging System (Universal Imaging, Downingtown, PA, USA).

### 3.3. Cresyl Violet Staining and TUNEL Assay

For histological experiments, slices were rinsed in PBS twice, fixed with cold 4% paraformaldehyde for 30 min, and stored in 30% sucrose at 4 °C overnight. Sections were cut with a cryostat at a thickness of 10 μm. Only sections from the middle to the upper part of the culture were used since these sections were similar. Cresyl violet staining was performed after 48 h of recovery time following KA treatment with ATPH or ATTN, and photomicrographs were taken at 10× magnification. The numbers of positive cells in CA3 region were counted using the MetaMorph Imaging system (Universal Imaging, Downingtown, PA, USA) by outlining the CA3 pyramidal cell layer. The mean value of three sections per slide for each group was used in different experiments. Apoptosis was assessed using the in situ apoptosis detection kit (ApopTag^®^ Plus Peroxidase In Situ Apoptosis Detection kit, Chemicon, Temecula, CA, USA). A TUNEL assay was performed 24 h after drugs exposure. Photomicrographs were taken at 10× magnification. Positive TUNEL staining was quantified in the same way as cresyl violet.

### 3.4. Evaluation of Intracellular ROS Formation

Formation of intracellular peroxides was detected using an oxidant-sensing fluorescent probe, 2′,7′-dichlorofluorescin diacetate (DCFH-DA, Sigma, St. Louis, MO, USA). DCFH-DA is cell-membrane permeable, and it is converted to 2′,7′-dichlorofluorescin (DCFH), which interacts with intracellular free radicals and peroxides to become fluorescent 2′,7′-dichlorofluorescein (DCF). 10 μM DCF-DA was applied to the culture medium at 35 °C for 30 min, and then the cultures were washed twice with phosphate-buffered saline (1× PBS, pH 7.4). The fluorescent DCF was detected using a fluorescence microscope (IX-71, Olympus, Tokyo, Japan), captured by a digital camera, and quantified using the MetaMorph Imaging System (Universal Imaging, Downingtown, PA, USA).

### 3.5. Lipid Peroxidation-TBARS Assay

Lipid peroxidation was measured using the OxiSelect™ TBARS Assay Kit (Cellbiolabs, San Diego, CA, USA) according to the manufacturer’s protocol. In brief, cultured slices were homogenized in a lysis buffer. Homogenates (100 μL) were mixed with a color reagent consisting of thiobarbituric acid (TBA), acetic acid and sodium hydroxide. The mixture was incubated at 95 °C for 1 h and centrifuged at 3000 rpm for 10 min. TBARS were measured by the absorbance at 532 nm. Data were calculated from MDA standard curve.

### 3.6. Statistical Analysis

Statistical calculations were performed using SPSS statistical program (SPSS Inc., Chicago, IL, USA). Results are expressed as mean ± S.E.M. Differences among groups were analyzed by Dunnett’s post-hoc test. The level of significance was accepted at *p* < 0.05.

## 4. Conclusions

The delayed and selective neuronal death in the CA3 area of OHSC after KA treatment was observed. KA treatment also caused the levels of DCF fluorescence and MDA to increase significantly. However, ATTN as well as ATPH prevented neuronal damage and reduced the ROS formation and lipid peroxidation compared to KA only-treated cultures. These results indicate that ATTN as a free radical scavenger such as ATPH has protective effects against cell death by KA in OHSC.
